# 1229. Antimicrobial Activity of Plazomicin against Multidrug-resistant *Enterobacterales*: Results from 3 Years of Surveillance in Hospitals in the United States (2018–2020)

**DOI:** 10.1093/ofid/ofab466.1421

**Published:** 2021-12-04

**Authors:** Cecilia G Carvalhaes, Jaideep Gogtay, Cheung Yee, Sandhya Das, Mariana Castanheira, Mariana Castanheira, Rodrigo E Mendes, Helio S Sader

**Affiliations:** 1 JMI Laboratories, Inc., North Liberty, Iowa; 2 Cipla Ltd., Mumbai, Maharashtra, India; 3 Cipla Therapeutics, Warren, New Jersey; 4 JMI Laboratories, North Liberty, IA

## Abstract

**Background:**

Multidrug-resistant (MDR) *Enterobacterales* isolates have increased and remain elevated in many US hospitals. Aminoglycoside (AMG) resistance often co-exist with resistance to other classes of antibiotics. A newer aminoglycoside, plazomicin, was evaluated against a large collection of MDR Enterobacterales clinical isolates from US hospitals.

**Methods:**

A total of 456 MDR isolates (1/patient) were collected from 32 US medical centers located in 23 states in 2018-2020 and susceptibility tested by broth microdilution method at a central laboratory. MDR was defined as nonsusceptible (NS) to ≥3 antimicrobial classes and extensively drug-resistant (XDR) as susceptible (S) to ≤2 classes. Isolates resistant to aminoglycosides and/or broad-spectrum cephalosporins were screened for aminoglycoside-modifying enzymes (AME), 16S rRNA methyltransferases, and β-lactamases by whole genome sequencing.

**Results:**

PLZ inhibited 93.0% of the MDR isolates (MIC_50/90_, 0.5/1 mg/L) and showed MIC values 8- to 16-fold lower than amikacin (AMK; MIC_50/90_, 4/16 mg/L; 93.2%S; Table). AMK S rates were 84.6% and 69.3% when EUCAST (≤8 mg/L) and USCAST (≤4 mg/L) breakpoints were applied, respectively. Among agents from other classes, S rates were 85.5% for meropenem, 88.4% for tigecycline, 49.3% for piperacillin-tazobactam, and 17.8% for cefepime; only the carbapenems and tigecycline were active against >50% of MDR isolates. PLZ retained activity against isolates NS to AMK (83.9%S), gentamicin (GEN; 89.3%S), and/or tobramycin (TOB; 92.4%S). PLZ showed markedly higher S rates than AMK against XDR (93.3% vs. 71.7%), AME producers (97.6% vs. 90.2%), and carbapenemase (CPE) producers (98.1% vs. 67.9%). PLZ was active against 99.0% of ESBL producers, while AMK S rates were 96.2%/87.0% as per the US FDA/EUCAST against these organisms. PLZ and AMK showed similar S rates when tested against GEN-NS isolates. GEN and TOB exhibited limited activity against MDR and all resistant subsets.

**Conclusion:**

Despite co-resistance to aminoglycosides and other classes of antibiotics observed with MDR *Enterobacterales* isolates, PLZ remained highly active against these isolates including AME-, ESBL-, and/or CPE-producers that cause infections in US hospitals.

Table

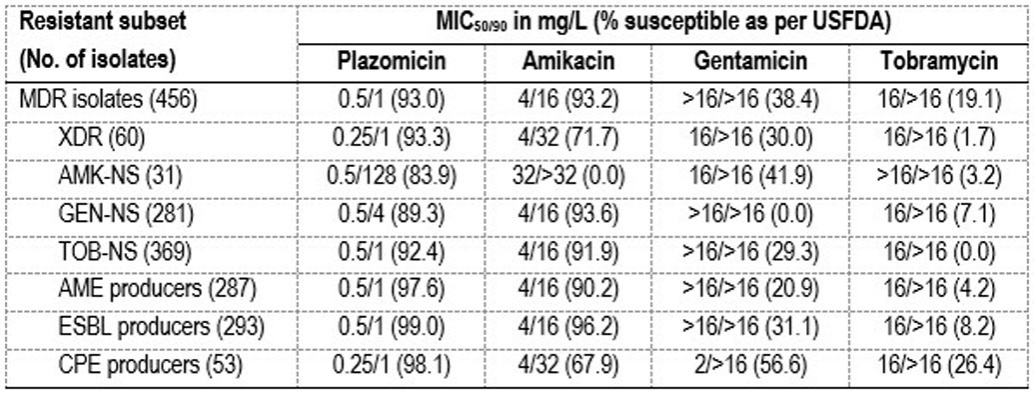

**Disclosures:**

**Cecilia G. Carvalhaes, MD, PhD**, **AbbVie (formerly Allergan**) (Research Grant or Support)**Cidara Therapeutics, Inc.** (Research Grant or Support)**Cipla Therapeutics** (Research Grant or Support)**Cipla USA Inc.** (Research Grant or Support)**Melinta Therapeutics, LLC** (Research Grant or Support)**Pfizer, Inc.** (Research Grant or Support) **Jaideep Gogtay, n/a**, **Cipla Therapeutics** (Employee)**Cipla USA Inc.** (Employee) **Cheung Yee, MSc, PhD**, **Cipla Therapeutics** (Employee) **Sandhya Das, n/a**, **Cipla Therapeutics** (Employee) **Mariana Castanheira, PhD**, **AbbVie (formerly Allergan**) (Research Grant or Support)**Bravos Biosciences** (Research Grant or Support)**Cidara Therapeutics, Inc.** (Research Grant or Support)**Cipla Therapeutics** (Research Grant or Support)**Cipla USA Inc.** (Research Grant or Support)**GlaxoSmithKline** (Research Grant or Support)**Melinta Therapeutics, Inc.** (Research Grant or Support)**Melinta Therapeutics, LLC** (Research Grant or Support)**Pfizer, Inc.** (Research Grant or Support)**Qpex Biopharma** (Research Grant or Support)**Shionogi** (Research Grant or Support)**Spero Therapeutics** (Research Grant or Support) **Mariana Castanheira, PhD**, Affinity Biosensors (Individual(s) Involved: Self): Research Grant or Support; Allergan (Individual(s) Involved: Self): Research Grant or Support; Amicrobe, Inc (Individual(s) Involved: Self): Research Grant or Support; Amplyx Pharma (Individual(s) Involved: Self): Research Grant or Support; Artugen Therapeutics USA, Inc. (Individual(s) Involved: Self): Research Grant or Support; Astellas (Individual(s) Involved: Self): Research Grant or Support; Basilea (Individual(s) Involved: Self): Research Grant or Support; Beth Israel Deaconess Medical Center (Individual(s) Involved: Self): Research Grant or Support; BIDMC (Individual(s) Involved: Self): Research Grant or Support; bioMerieux Inc. (Individual(s) Involved: Self): Research Grant or Support; BioVersys Ag (Individual(s) Involved: Self): Research Grant or Support; Bugworks (Individual(s) Involved: Self): Research Grant or Support; Cidara (Individual(s) Involved: Self): Research Grant or Support; Cipla (Individual(s) Involved: Self): Research Grant or Support; Contrafect (Individual(s) Involved: Self): Research Grant or Support; Cormedix (Individual(s) Involved: Self): Research Grant or Support; Crestone, Inc. (Individual(s) Involved: Self): Research Grant or Support; Curza (Individual(s) Involved: Self): Research Grant or Support; CXC7 (Individual(s) Involved: Self): Research Grant or Support; Entasis (Individual(s) Involved: Self): Research Grant or Support; Fedora Pharmaceutical (Individual(s) Involved: Self): Research Grant or Support; Fimbrion Therapeutics (Individual(s) Involved: Self): Research Grant or Support; Fox Chase (Individual(s) Involved: Self): Research Grant or Support; GlaxoSmithKline (Individual(s) Involved: Self): Research Grant or Support; Guardian Therapeutics (Individual(s) Involved: Self): Research Grant or Support; Hardy Diagnostics (Individual(s) Involved: Self): Research Grant or Support; IHMA (Individual(s) Involved: Self): Research Grant or Support; Janssen Research & Development (Individual(s) Involved: Self): Research Grant or Support; Johnson & Johnson (Individual(s) Involved: Self): Research Grant or Support; Kaleido Biosceinces (Individual(s) Involved: Self): Research Grant or Support; KBP Biosciences (Individual(s) Involved: Self): Research Grant or Support; Luminex (Individual(s) Involved: Self): Research Grant or Support; Matrivax (Individual(s) Involved: Self): Research Grant or Support; Mayo Clinic (Individual(s) Involved: Self): Research Grant or Support; Medpace (Individual(s) Involved: Self): Research Grant or Support; Meiji Seika Pharma Co., Ltd. (Individual(s) Involved: Self): Research Grant or Support; Melinta (Individual(s) Involved: Self): Research Grant or Support; Menarini (Individual(s) Involved: Self): Research Grant or Support; Merck (Individual(s) Involved: Self): Research Grant or Support; Meridian Bioscience Inc. (Individual(s) Involved: Self): Research Grant or Support; Micromyx (Individual(s) Involved: Self): Research Grant or Support; MicuRx (Individual(s) Involved: Self): Research Grant or Support; N8 Medical (Individual(s) Involved: Self): Research Grant or Support; Nabriva (Individual(s) Involved: Self): Research Grant or Support; National Institutes of Health (Individual(s) Involved: Self): Research Grant or Support; National University of Singapore (Individual(s) Involved: Self): Research Grant or Support; North Bristol NHS Trust (Individual(s) Involved: Self): Research Grant or Support; Novome Biotechnologies (Individual(s) Involved: Self): Research Grant or Support; Paratek (Individual(s) Involved: Self): Research Grant or Support; Pfizer (Individual(s) Involved: Self): Research Grant or Support; Prokaryotics Inc. (Individual(s) Involved: Self): Research Grant or Support; QPEX Biopharma (Individual(s) Involved: Self): Research Grant or Support; Rhode Island Hospital (Individual(s) Involved: Self): Research Grant or Support; RIHML (Individual(s) Involved: Self): Research Grant or Support; Roche (Individual(s) Involved: Self): Research Grant or Support; Roivant (Individual(s) Involved: Self): Research Grant or Support; Salvat (Individual(s) Involved: Self): Research Grant or Support; Scynexis (Individual(s) Involved: Self): Research Grant or Support; SeLux Diagnostics (Individual(s) Involved: Self): Research Grant or Support; Shionogi (Individual(s) Involved: Self): Research Grant or Support; Specific Diagnostics (Individual(s) Involved: Self): Research Grant or Support; Spero (Individual(s) Involved: Self): Research Grant or Support; SuperTrans Medical LT (Individual(s) Involved: Self): Research Grant or Support; T2 Biosystems (Individual(s) Involved: Self): Research Grant or Support; The University of Queensland (Individual(s) Involved: Self): Research Grant or Support; Thermo Fisher Scientific (Individual(s) Involved: Self): Research Grant or Support; Tufts Medical Center (Individual(s) Involved: Self): Research Grant or Support; Universite de Sherbrooke (Individual(s) Involved: Self): Research Grant or Support; University of Iowa (Individual(s) Involved: Self): Research Grant or Support; University of Iowa Hospitals and Clinics (Individual(s) Involved: Self): Research Grant or Support; University of Wisconsin (Individual(s) Involved: Self): Research Grant or Support; UNT System College of Pharmacy (Individual(s) Involved: Self): Research Grant or Support; URMC (Individual(s) Involved: Self): Research Grant or Support; UT Southwestern (Individual(s) Involved: Self): Research Grant or Support; VenatoRx (Individual(s) Involved: Self): Research Grant or Support; Viosera Therapeutics (Individual(s) Involved: Self): Research Grant or Support; Wayne State University (Individual(s) Involved: Self): Research Grant or Support **Rodrigo E. Mendes, PhD**, **AbbVie** (Research Grant or Support)**AbbVie (formerly Allergan**) (Research Grant or Support)**Cipla Therapeutics** (Research Grant or Support)**Cipla USA Inc.** (Research Grant or Support)**ContraFect Corporation** (Research Grant or Support)**GlaxoSmithKline, LLC** (Research Grant or Support)**Melinta Therapeutics, Inc.** (Research Grant or Support)**Melinta Therapeutics, LLC** (Research Grant or Support)**Nabriva Therapeutics** (Research Grant or Support)**Pfizer, Inc.** (Research Grant or Support)**Shionogi** (Research Grant or Support)**Spero Therapeutics** (Research Grant or Support) **Helio S. Sader, MD, PhD, FIDSA**, **AbbVie (formerly Allergan**) (Research Grant or Support)**Basilea Pharmaceutica International, Ltd.** (Research Grant or Support)**Cipla Therapeutics** (Research Grant or Support)**Cipla USA Inc.** (Research Grant or Support)**Department of Health and Human Services** (Research Grant or Support, Contract no. HHSO100201600002C)**Melinta Therapeutics, LLC** (Research Grant or Support)**Nabriva Therapeutics** (Research Grant or Support)**Pfizer, Inc.** (Research Grant or Support)**Shionogi** (Research Grant or Support)**Spero Therapeutics** (Research Grant or Support)

